# Somatotype of Top-Level Serbian Rhythmic Gymnasts

**DOI:** 10.2478/hukin-2014-0020

**Published:** 2014-04-09

**Authors:** Tijana Purenović-Ivanović, Ružena Popović

**Affiliations:** 1 University of Niš, Faculty of Sport and Physical Education, Serbia.

**Keywords:** rhythmic gymnastics, selection, kinanthropometry, constitution, elite athletes

## Abstract

Body size and build influence performance in many sports, especially in those belonging to the group of female aesthetic sports (rhythmic gymnastics, artistic gymnastics, and figure skating). These sports pose high specific demands upon the functional, energy, motor and psychological capacities of athletes, but also upon the size, body build and composition of the performers, particularly of the top-level female athletes. The study of the top athletes (rhythmic gymnasts, in this case) may provide valuable information on the morphological requirements for achieving success in this sport. Therefore, the main objective of this research was to analyze the somatotype of 40 Serbian top-level rhythmic gymnasts, aged 13.04±2.79, and to form the five age group categories. The anthropometric variables included body height, body mass, the selected diameters, girths and skinfolds, and the Heath-Carter anthropometric somatotype. All of the anthropometric data were collected according to International Biological Programme, and then processed in the Somatotype 1.2. The applied analysis of variance indicated an increase in endomorphic component with age. The obtained results show that the balanced ectomorph is a dominant somatotype, being similar for all of the athletes that took part in the research (3.54-3.24-4.5). These results are in line with the ones obtained in previous studies.

## Introduction

Athletes are characterized by combination of body composition/body size traits which are believed to influence the chance of success in any given sport.Therefore, it is suggested that the measurement of kinanthropometry is a crucial tool in the search for information that would assist coaches and athletes in the quest for success at the highest level in sport, i.e. in talent identification (Sánchez-Muñoz et al., 2012).

Literature describes desirable model characteristics of the elite athletes in the form of basic anthropometric dimensions, their interrelations, body composition components, and somatotypes. Body size and body build contribute significantly to performance in many sports, particularly in aesthetic sports (Misigoj-Durakovic, 2012). Within the group of the so-called female aesthetic sports, the rhythmic gymnastics (RG) is one of the most demanding.

This discipline imposes high demands upon body size, body build and composition of the performers, particularly of elite female athletes. A vast majority of the authors (Lapieza et al., 1993;Menezes and Filho, 2006; Amigo et al., 2009;Poliszczuk and Broda, 2010; Quintero et al., 2011) have proposed one, the most common model of the somatotype that rhythmic gymnasts (RGs) pertain to - the balanced ectomorph - which implies that the ectomorphic component is the dominant one and the remaining two have equal prevalence. However, there are some other models which should not be overlooked, such as the mesoectomorph (Lopéz-Benedicto et al., 1991;Amigo et al., 2009), the mesomorph-ectomorph (Vernetta et al., 2011), as well as the central somatotype (Quintero et al., 2011; Vernetta et al., 2011), and even the balanced endomorph (Quintero et al., 2011).

Taking into account the fact that the relevant literature has not established one morphological prototype of rhythmic gymnasts as the dominant one and the fact that there are no relevant data on the somatotype of Serbian RGs, the purpose of the present study was to assess the body constitution of the top-level rhythmic gymnasts in Serbia. The main objective of the study was to determine somatic characteristics of Serbian girls of different age who were competing at the top-level RG and thus provide valuable information for the future selection process.

## Material and Methods

### Participants

Forty rhythmic gymnasts (age: 13.04±2.79 years, body height: 153.64±13.16 cm, body mass: 40.55±11.31 kg) volunteered to participate in the study. A written request was promptly sent to the Expert Committee of Gymnastics Federation of Serbia, and, after being informed about the study, its scientific value and multiple benefits, the approval was given for the testing to be conducted during the 2012 National Championships. All clubs participating in the National Championships were informed about the conducted research and four out of ten RG clubs have given their consent. All participants are an “A” program, group routines competitors. The baseline characteristics of the sample in total, and sub-samples (age categories) are presented in Table 1.

### Measures and Procedures

The measures were taken during 2012 National Championships held in Belgrade (Serbia) on December 16^th^ 2012, and testing was conducted in agreement with the principles stated in the Declaration of Helsinki (WMA, 2002). An anthropometric method was used for obtaining the RGs’ somatic type and it included 10 following variables: body height (in cm), body mass (in kg), four skinfolds (over triceps, subscapular, supraspinale, and calf; in mm), and biceps girth (flexed 90° and tensed; in cm), standing calf girth (in cm), humerus breadth (in cm) and femur breadth (in cm).

All of the measurements were taken by both authors in the optimal climatic conditions, with the participants in underwear, and according to the methods proposed by the International Biological Programme (Weiner and Lourie, 1969). The body mass was measured with a digital scale Omron BF511 (Kyoto, Japan).

### Analysis

The somatotype was determined according to the methodology of Heath-Carter (Carter and Heath, 1990), applying the statistical data analysis (Descriptive statistics and one-way ANOVA) using the computer program *Somatotype 1.2*.

## Results

The obtained data are presented in tables and graphs (by somatoplots). The sample of 40 top-level Serbian RGs showed that the mean somatotype was: 3.54 - 3.24 - 4.5 (values for the endomorphy, mesomorphy and ectomorphy, respectively; Table 2). The descriptive statistics of all the measurements are presented in Table 2, and all the forty profiles (squares) with the mean somatotype (circle) are presented in Figure 2.

**Figure 1.**
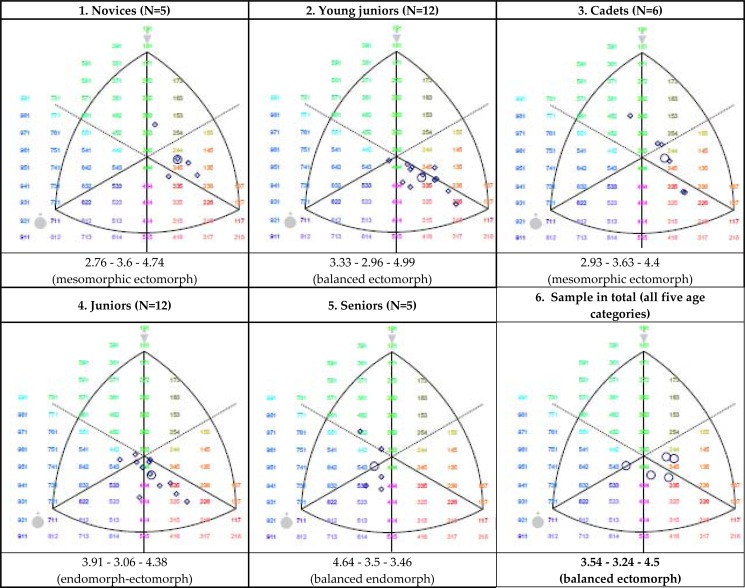
Somatoplots of top-level Serbian RGs, according to five age categories. The squares are the individual somatotypes, and the circle is the mean profile.

**Figure 2.**
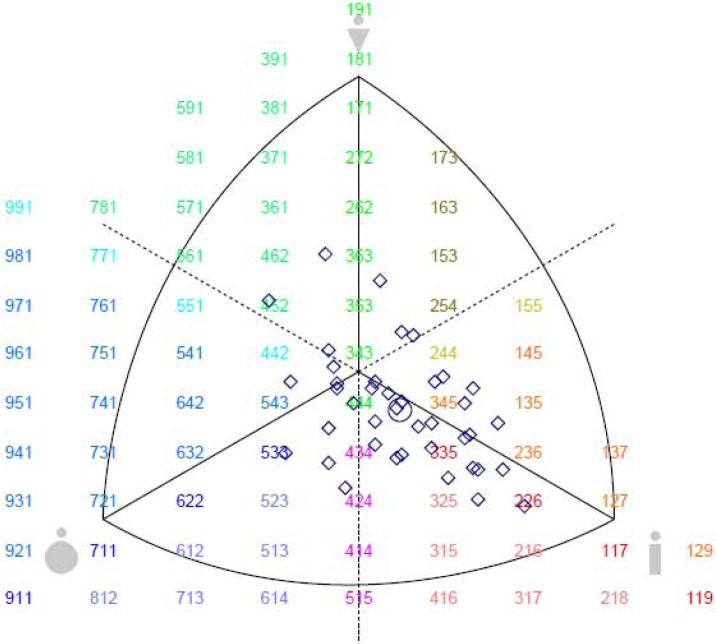
Somatotype distribution of top-level Serbian RGs (N=40). The squares are the individual somatotypes, and the circle is the mean profile.

Analyzing the somatoplots presented in Figure 1, a variety of somatotype categories can be noticed, with one major category (the ectomorphy) prevailing. It is obvious that an increase in age entails an increase in the value of the endomorphic component while the other two components remain rather stable. This is confirmed by ANOVA due to the presence of statistically significant differences (F=3.6, p=0.015) among the five age categories. The discriminatory factor (apart from the age, height and body mass) was one somatotype component (endomorphy, p<0.001). Such results are common in many other sports. What can be said about the range of RGs’ somatotype components is that they are moderate.

## Discussion

Apart from talent, the adequate body constitution is a prerequisite for achieving success in sports. Body build is, to a large extent, determined by the human genotype, but within the defined limits it is also subject to environmental influence. The extent of sensitivity to the external environment is also hereditary conditioned.

The type of body constitution in the observed RGs was mainly characterized by the prevalence of the ectomorphic component, with moderate values of the other two components (3.54-3.24-4.5). This balanced ectomorph constitution is seen in other RG studies. For example, Lapieza et al. (1993) analyzed the somatotype of 18 female rhythmic gymnasts from Spain, aged 12 to 16 (belonging to cadet and junior teams) and achieved the following mean profile: 2.28–2.45–3.7. What can be noticed are the low values of the first and the second component, which are, at the same time, lower than the values we obtained from our cadet team gymnasts (2.93-3.63-4.4), as well as the junior team gymnasts (3.91-3.06-4.38). Menezes and Filho (2006) analyzed the somatotype of 24 female Brazilian rhythmic gymnasts (seven from the National RG Team, 10 participants from the 2003 National Championships, and seven participants from the Regional Championships from Rio de Janeiro), and obtained the following profiles: 2.33-2.83-4.17, 2.72-2.65-4.17 and 2.88-3.16-3.51. Even here the low values of the first two components are noticeable as was the case with the previously mentioned study. Poliszczuk and Broda (2010) analyzed 19 female rhythmic gymnasts, aged 8 to 11 and found the following somatotype: 2.65-2.45-3.95, which, when compared to the somatotype of our novices (2.76-3.6-4.74), shows the low values of the mesomorphic and the ectomorphic components.

Lopéz-Benedicto et al. (1991) in the sample of 21 Spanish junior RGs of a regional and national level (11.1 to 15.8 ages), found the mesoectomorph somatotype profile (2.3-3.1-4.5) as the result of their study. Vernetta et al. (2011) did their research with 20 Andalusian RGs, 9 to 15 years of age, and divided them into two sub-samples: A(N=12): 9–11 years of age (1.804-3.694-3.701, mesomorphic ectomorph) and B(N=8): 12–15 years of age (2.059-3.161-4.115, central somatotype). The total sample showed a central mean somatotype (2.906-3.481-3.866).

Amigo et al. (2009) (N=151 RGs, aged 10–18) and Quintero et al. (2011) (2008 Tenerife Championships, N=70 RGs, aged 8–19) conducted longitudinal studies with national and international level Spanish RGs (results are presented in Table 3) and obtained quite different results, which can be noted when the somatotype components’ values of the gymnasts of the same age are compared. Namely, Spanish gymnasts from the study by Amigo et al. (2009) are of great somatotype stability across ages, and they have the mesoectomorphic and balanced ectomorph body constitution, i.e. very low endomorphic and mesomorphic component values and very high ectomorphic values. In the second study (Quintero et al., 2011), one can notice the moderate values for all of the three somatotype components, but with an endomorphic value as the highest one (an increase of the endomorphic component with age). However, in the study by Quintero et al. (2011), authors did not specify the competition level of studied RGs, which could be possible explanation for the discrepancies between the obtained results of these two studies.

## Conclusions

All of the previous research results mostly indicate graceful and thin (ectomorphic) body build of rhythmic gymnasts, characterized by long, slim and thin limbs, with small circumferences of muscles and thin and light bones, made for subtle and graceful movements. However, top-level Serbian RGs can be characterized as balanced ectomorph, with not so low values for endomorphic and mesomorphic components, which is not seen as consistent with the somatotype of RGs from other countries (the ones that have higher ranking in rhythmic gymnastics). Apart from that, the differences amongst the five age group categories of the top-level Serbian RGs were established, and they indicate that the endomorphic component increases with age, while the other two components are rather stable (this could be due to maturation, or even genetics and nutrition factor). Having also in mind the fact that there is no evidence of this two-way causality between the body constitution and sport (is the somatic type a cause or a consequence?), this interpretation of our findings still does not represent sufficient information for the talent identification process in RG. It could, however, inform about where the Serbian RG was positioned in relation to more successful RG countries.

## Study Limitations

Having in mind the fact that results in sports do not depend solely on the physique of an athlete, at this point we can detect the main flaw of the study: it does not take into consideration other abilities that influence performance in sports (such as motor abilities, psychological state, functional abilities, etc.). Another area that could be improved would be the sample of participants and a study design type, which hinders the authors from more significant conclusions. Maturity, of which the tempo and the period of time of its occurrence varies and is associated with an increase of the body height and body mass, could also represent the key points for a variety of body constitution types. Therefore, it is recommended that the status of the Serbian rhythmic gymnasts should undergo multidisciplinary analysis on the basis of a larger and more representative sample, monitored over a period of few years.

## Figures and Tables

**Table 1:** The baseline characteristics of top-level Serbian RGs

**Age Categories**	**Variable**	**Mean±SD**	**Median**	**Range**
**Novices (N=5)**	Age	8.99±1.16	8.93	8.07 – 10.92
	Body Height	133.32±6.17	133	127.2 – 142.5
	Body Mass	25.3±3.4	24.7	22.3 – 30.8
**Young juniors (N=12)**	Age	11.07±0.73	11.17	9.94 – 11.92
	Body Height	145.64±7.81	145.9	134 – 158.4
	Body Mass	32.37±5.65	31.3	25.9 – 44.5
**Cadets (N=6)**	Age	12.84±0.65	12.9	12 – 13.57
	Body Height	152.97±7.36	151.25	143.7 – 164
	Body Mass	39.27±4.23	39.35	33.7 – 44.3
**Juniors (N=12)**	Age	14.51±0.78	14.24	13.48 – 16.59
	Body Height	164.09±4.27	163.1	158.5 – 171
	Body Mass	48.63±4.64	48.05	41.3 – 56.3
**Seniors (N=5)**	Age	18.13±1.18	17.57	16.93 – 19.45
	Body Height	168.88±6.22	168.2	161 – 175.1
	Body Mass	57.6±3.69	56.2	54 – 61.6
**Total (N=40)**	Age	13.04±2.79	12.9	8.07 – 19.45
	Body Height	153.64±13.16	156.75	127.2 – 175.1
	Body Mass	40.55±11.31	40.4	22.3 – 61.6

**N** – number of study participants, SD – standard deviation. Age is presented in years, body height in cm, and body mass in kg.

**Table 2:** The descriptive statistics of the measured anthropometric variables of the sample in total (N=40)

***Variable***	***Mean*±*SD***	***Median***	***Range***
***Triceps SF***	12.74±3.87	11.9	6 – 21.2
***Subscapular SF***	9±2.48	8.5	4.2 – 14.2
***Supraspinale SF***	10.03±3.74	9.6	4.2 – 18.8
***Calf SF***	8.79±3.81	8.1	3 – 17.8
***Flexed arm G***	22.95±2.68	23.1	17.5 – 28.2
***Calf G***	30.91±3.62	30.8	24 – 38.1
***Humerus B***	6.11±0.6	6.17	4.75 – 7.02
***Femur B***	7.84±0.7	8	6.53 – 8.82
***HWR***	45.17±1.23	45.05	42.41 – 48
***SAD***	1.32±0.68	1.32	0.08 – 2.89
***Endomorphy***	3.54±0.82	3.5	2.1 – 5.3
***Mesomorphy***	3.24±0.86	3.2	1.8 – 5.5
***Ectomorphy***	4.5±0.91	4.4	2.5 – 6.6

SF – skinfold, G – girth, B – breadth, HWR – height-weight ratio, SAD – somatotype attitudinal distance, SD – standard deviation.

**Table 3:** The evolution of somatotype with age (Spanish RGs)

**Study**	**Age**	**N**	**Endo**	**Meso**	**Ecto**
**Amigo et al. (2009)**	10	12	1.4	2.6	5.5
	11	11	1.4	2.5	5.4
	12	15	1.4	2.4	5.5
	13	23	1.5	2.4	5.5
	14	27	1.6	2.7	5.2
	15	25	1.7	2.5	5.1
	16	14	1.7	2.6	4.8
	17	14	1.9	2.7	4.8
	18	10	1.8	2.6	4.9
**Quintero et al.(2011)**	8 – 11	12	4.25	3.28	4.8
	11 – 13	11	4.13	3.01	4.64
	13 – 14	23	4.59	3.06	3.89
	15 – 19	25	4.45	3.33	3.57

N – number of study participants, Endo – endomorphy, Meso – mesomorphy, Ecto – ectomorphy.

Age is presented in years.
